# Comparative CBCT Analysis of Maxillofacial Skeletal Structures in Patients with Unilateral Cleft Lip and Palate and Non-Cleft Individuals

**DOI:** 10.3390/diagnostics14222555

**Published:** 2024-11-14

**Authors:** Emre Haylaz, Fahrettin Kalabalık, Orhan Cicek, İsmail Gümüşsoy, Emre Aytuğar

**Affiliations:** 1 Department of Oral and Maxillofacial Radiology, Faculty of Dentistry, Sakarya University, Sakarya 54100, Turkey; fahrettinkalabalik@sakarya.edu.tr (F.K.); ismailgumussoy@sakarya.edu.tr (İ.G.); 2 Department of Orthodontics, Faculty of Dentistry, Zonguldak Bülent Ecevit University, Zonguldak 67600, Turkey; orhancicek@beun.edu.tr; 3 Department of Oral and Maxillofacial Radiology, Faculty of Dentistry, Izmir Katip Celebi University, İzmir 35640, Turkey; emreaytugar@gmail.com

**Keywords:** cleft lip and palate, craniofacial anomalies, cone beam computed tomography

## Abstract

Background: The aim of this study was to evaluate and compare the maxillofacial structures of individuals with unilateral cleft lip and palate (UCLP) and healthy controls using cone beam computed tomography (CBCT). Methods: The study included a total of 90 subjects, comprising 45 randomly selected individuals with UCLP (30 males and 15 females, mean age 14.69 ± 3.95 years) in the study group and 45 healthy individuals (30 males and 15 females, mean age 14.46 ± 3.65 years) in the control group. Maxillofacial measurements were taken in three different planes and categorized into five groups, namely vertical, facial, cranial, maxillary, and mandibular. In the statistical comparison between groups, the significance level was determined as *p* < 0.05. Results: There were no significant differences in the age and gender distributions between the groups (*p* > 0.05). Upper anterior face height and posterior face height in the UCLP group were found to be significantly shorter than the control group (*p* < 0.05). Midface width and depth were inadequate in the UCLP group (*p* < 0.05). Anterior and posterior cranial base lengths were significantly shorter in individuals with UCLP (*p* < 0.05). Nasal width and interorbital width were significantly greater in the UCLP group (*p* < 0.05). In addition, maxillary width, maxillary length, and mandibular width were significantly shorter in the UCLP group than in the control group (*p* < 0.05). Conclusions: While the control group exhibited generally longer measurements in all three dimensions compared to the study group, the skeletal structures adjacent to the cleft demonstrated the most notable developmental deficiency.

## 1. Introduction

Patients born with cleft lip and palate (CLP) frequently encounter both functional and aesthetic challenges, necessitating repeated surgical procedures throughout their growth and development [1]. Unilateral cleft lip and palate (UCLP) presents a persistent challenge for patients, parents, and cleft care professionals, requiring multiple revisions, fistula repairs, bone grafting, and orthodontic follow-up after the initial surgery [2]. Therefore, cleft or reconstructive surgery may be responsible for the insufficiency of maxillofacial growth in individuals with clefts [3]. The necessity for surgical intervention should be clarified through a careful evaluation of the patients’ functional facial growth and development [1,4]. It is critical for patients with UCLP to achieve normal functional, intellectual, and psychological development not only through surgical methods but also through non-surgical approaches, such as speech therapy, psychological support, and orthodontic treatment [5]. Therefore, it is evident that the care of UCLP patients requires long-term and continuous follow-up through a multidisciplinary team approach, involving professionals from several fields at each stage of growth.

The etiology of CLP, caused by the failure of fusion of the maxillary and palatal processes, is complex and involves multiple risk variables, including diabetes, folic acid deficiency, maternal age, smoking, and drug use during pregnancy as well as both genetic and environmental factors [6,7]. Based on the anatomical location and type, clefts may present as lip, palate, or both, and can be classified as either complete or incomplete, as well as unilateral or bilateral [6]. It may also develop as syndromic or non-syndromic depending on the presence of other physical and developmental malformations in affected individuals [8]. Although 70% of individuals with UCLP are non-syndromic, only 50% of isolated cleft palates may occur with any syndrome [9].

It has been reported that intrinsic or teratogenic factors may also play a role in the embryonic stage of maxillofacial developmental deficiencies, which are observed as a result of maxillary developmental deficiencies in patients with CLP [10]. In addition, it is well known that iatrogenic scar tissue after surgery for UCLP negatively affects maxillofacial growth and causes midface hypoplasia [11]. Therefore, in addition to maxillary constriction and hypoplasia, maxillary vertical deficiency, cranial base flexure, increased gonial angle, mandibular posterior rotation, and increases in lower anterior facial height can also be observed in patients with UCLP [12].

In the literature, lateral cephalometric radiographs are commonly used for craniofacial skeletal assessment and growth follow-up in patients with CLP [12,13,14]. On the other hand, the advent of cone beam computed tomography (CBCT) has enabled a three-dimensional and more comprehensive evaluation of the maxillomandibular skeleton in individuals with UCLP [15,16,17]. In this context, considering that individuals with UCLP are more likely to deviate from normal growth and development due to functional, environmental, and genetic factors [17], clarifying the differences between the maxillofacial skeletal structures of patients with UCLP who have not received orthodontic treatment and non-cleft healthy individuals is inevitable for an accurate multidisciplinary therapy approach. Thus, investigating maxillofacial skeletal differences in the transverse, sagittal, and vertical dimensions between individuals with UCLP and non-cleft individuals will not only provide an accurate diagnosis for subsequent surgically supported or non-surgical orthopedic, orthodontic, and prosthetic rehabilitation but also offer clinicians valuable insights into achieving functionally stable occlusal outcomes.

Therefore, this study aimed to investigate the transverse, sagittal, and vertical maxillofacial skeletal differences between patients with UCLP and healthy individuals without clefts using CBCTs. The alternative hypothesis (H_1_) of the study posits that differences exist between the groups across all of the parameters measured in the transverse, sagittal, and vertical dimensions.

## 2. Materials and Methods

### 2.1. Study Design and Ethical Approval

This retrospective study was conducted using the CBCT archive records of patients who had been referred to the Department of Oral and Maxillofacial Radiology, Izmir Katip Çelebi University, Faculty of Dentistry prior to orthodontic treatment. Ethical approval for this study was granted by the Non-Interventional Clinical Research Ethics Committee of Izmir Katip Çelebi University (Approval No: 0122/2024) prior to its commencement.

### 2.2. Sample Size and Criteria

The required sample size was determined through a power analysis using G*Power software (version 3.1.9.7; Franz Faul, Universität Kiel, Kiel, Germany) and based on the study by Ayub et al. [18]. Accordingly, if the α error probability is set at 0.05 and the study power (1 − β error probability) is set at 0.95, it was calculated that the actual power of the study would be 95% (actual power: 0.9527318) with a minimum of 80 samples (40 samples per group) included, with a noncentrality parameter δ = 3.6775488 and a critical t = 1.9908471. To further increase the power of the study, CBCT images from 45 subjects were included in each group. The study group consisted of 45 subjects with UCLP (30 males and 15 females). CBCT images of 45 age- and gender-matched subjects without clefts (30 males and 15 females) were included in the study as a control group.

The inclusion criteria for the study group are as follows:

- individuals with complete unilateral cleft lip and palate;
- individuals who underwent primary lip repair before the average age of one year;
- individuals who had a hard palate repair before the age of three but did not undergo additional surgery thereafter.

The inclusion criteria for the control group are as follows:

- being within the same age range as the study group;
- being a healthy individual with normal growth and development.

The exclusion criteria are as follows:

- having received prior orthodontic treatment;
- having a history of any syndrome or trauma;
- incomplete growth and development;
- CBCT images with poor/low image quality.

### 2.3. Obtaining and Evaluating CBCT Images

**Figure 1:**
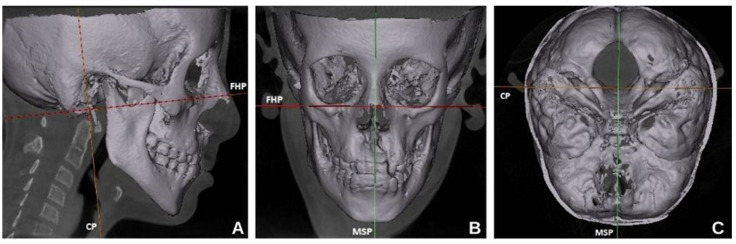
CBCT images with the orthogonal planes. FHP—Frankfurt horizontal plane (**A**), MSP—midsagittal plane (**B**), CP—coronal plane (**C**).

The Cone beam computed tomography (CBCT) images were acquired using a NewTom 5G (QR s.r.l., Verona, Italy), large fields of view (15 × 12 cm and 18 × 16 cm), of view 1–20 mA, and 110 kVp. The images were evaluated under dim lighting conditions using a medical monitor (Eizo Co.; Ishikawa, Japan) and the NNT program v.11.5 for Windows (QR Verona, s.r.l.; Verona, Italy). The images obtained with CBCT in the DICOM (Digital Imaging and Communication in Medicine) format were transferred to Mimics Materialize 20.0^®^ (Materialise, Leuven, Belgium) software for three-dimensional evaluation and linear measurements. The measurements on CBCT images were made on axial and coronal sections using a digital ruler. Axis orientations were made on axial, coronal, and sagittal section images for standardization of the lengths to be measured (Figure 1).

**Figure 2:**
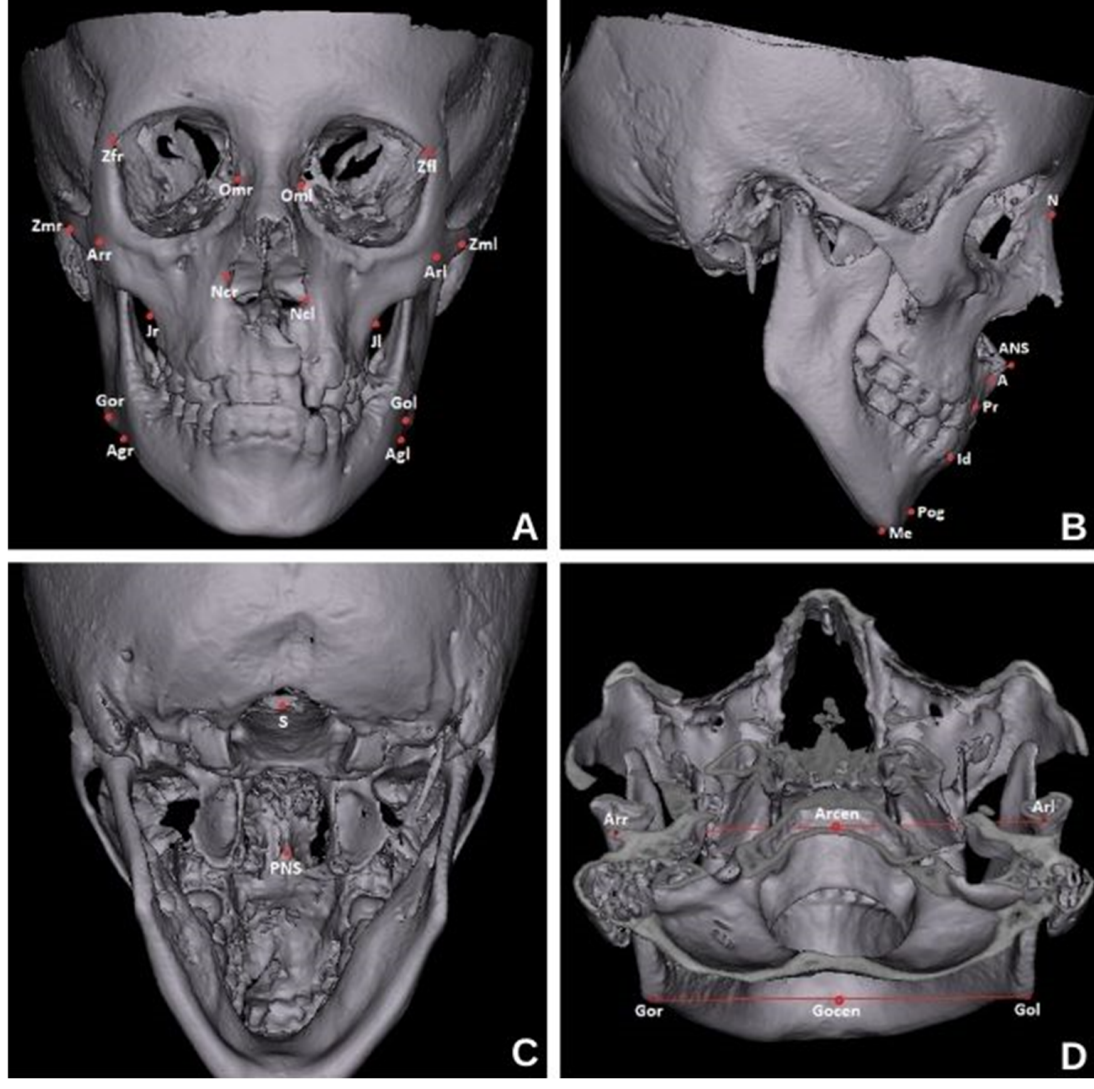
Anatomical points used for linear measurements in coronal (**A**,**C**), sagittal (**B**), and axial (**D**) sections.

Unilateral and bilateral anatomical points used in the study were marked on the reconstructed CBCT images (Figure 2).

**Figure 3:**
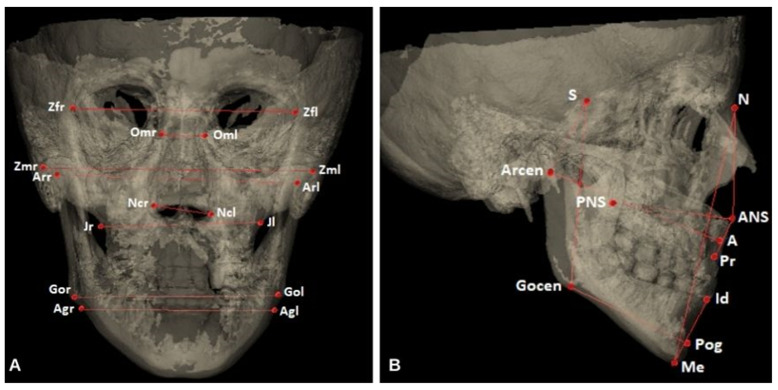
Linear measurements in coronal (**A**) and sagittal (**B**) sections in transparent 3D images.

The CBCT measurements were categorized into 5 groups, namely vertical, facial, cranial, maxillary, and mandibular, and the measurements were calculated in millimeters (mm). In Figure 3, the transparency of the CBCT reconstruction image was increased with the help of the program, and the length measurements are shown. The definitions of the reference planes and anatomical points evaluated in the study are given in Table 1.

### 2.4. Statistical Analysis

Statistical analyses of the data were performed using SPSS v.22.0 for Windows (IBM, Chicago, IL, USA). For the quantitative data found to be normally distributed by the Kolmogorov–Smirnov test, the *t*-test was used for pairwise comparisons. Chi-square tests were applied for categorical variables. Reliability for repeated measurements was assessed using intraclass correlation coefficients (ICCs). Accordingly, high reliability with ICCs of at least 0.92 was found for all measurements taken by the same investigator (EH) on 24 randomly selected CBCT images with a 4-week interval. The statistical significance was set at *p* < 0.05.

## 3. Results

In this study, the archived CBCT images of a total of 90 individuals (30 females and 60 males), consisting of 45 with UCLP in the study group and 45 healthy individuals in the control group, were retrospectively examined. The ages of all individuals included in the study ranged between 9 and 24.96 years, and the mean age was found to be 14.57 ± 3.78 years. The ages of the individuals in the study group included in the study ranged between 9 and 24.96 years, and the mean age was determined as 14.69 ± 3.95 years. The age mean of the control group was between 9.38 and 24.58 years, and the mean age was determined as 14.46 ± 3.65 years. When the gender and age distributions were examined between the study and control groups, it was determined that there was no significant difference (*p* > 0.05). The descriptive statistics results, including the study and control groups, are given in Table 2.

When the vertical dimension variables between the control group and the study group were compared, it was determined that the upper anterior facial height (N-ANS) and posterior facial height (S-Go_cen_) were significantly higher in the control group (*p* < 0.05). While the total anterior facial height (N-Me) was higher in the control group, the lower anterior facial height (ANS-Me) was higher in the UCLP group. However, in the comparison between the groups, the difference between the N-Me and ANS-Me variables was insignificant (*p >* 0.05). These results show that the upper facial height and posterior facial height in the UCLP patients are significantly shorter than in healthy controls (Table 3).

When the measurements regarding the facial profile were examined, facial width (Zm_r_-Zm_l_) and facial depth (Ar_cen_-A) were found to be significantly higher in the control group than in the study group (*p* < 0.05). On the other hand, nasal width (Nc_r_-Nc_l_) and interorbital (Om_r_-Oml) width were found to be significantly higher in the study group *(p* < 0.05). The biorbital width (Zf_r_-Zf_l_) does not show a statistically significant difference between the groups (*p* > 0.05). These results show that UCLP patients have a facial profile with a narrower face, wider nose, and larger medial orbital width than the control group. The anterior cranial base (S-N) and posterior cranial base (S-Ar_cen_) measurements, which are parameters related to deep structures, were determined to be significantly higher in the control group than in the study group (*p* < 0.05) (Table 3).

Considering the maxillary measurements, ANS-PNS (maxillary length) and J_r_-J_l_ (maxillary width) values were determined to be significantly higher in the control group (*p* < 0.05). No significant difference was detected between the groups for the maxillary anterior alveolar height (ANS-Pr) measurement (*p* > 0.05). For the mandibular measurements, only the mandibular width (Ag_r_-Ag_l_) was stated to be significantly higher in the control group than in the study group (*p* < 0.05). No statistically significant difference was detected in the comparison between groups for the mandibular length (Go_cen_-Pog) and mandibular anterior alveolar height (Id-Me) measurements (*p* > 0.05) (Table 3). These results show that, in an intergroup comparison, individuals with UCLP have shorter palatal length and narrower maxillary and mandibular width.

## 4. Discussion

Craniofacial growth and development is not merely a two-dimensional event. Rather, it is a process that occurs in three dimensions and yields different outcomes across various planes. Therefore, the selected research method should be of a nature that aligns with this characteristic of growth. Craniofacial measurements can be influenced by numerous variables. Therefore, it has been reported that cephalometric analyses are inadequate for assessing three-dimensional structures in a two-dimensional context. It has been reported that the abnormal facial morphology and distortion observed in individuals with CLP present challenges to identifying certain cephalometric landmarks, necessitating careful interpretation of the results of cephalometric studies. A study conducted solely on lateral cephalometric radiographs can shed light on only a limited aspect of craniofacial development [19,20,21]. There are very few CBCT studies in the literature that compare the maxillofacial morphology between UCLP and healthy individuals [16]. For this reason, this study employed CBCT to compare the maxillofacial skeletal characteristics of individuals with UCLP who underwent the same surgical operation with age- and gender-matched individuals exhibiting normal growth patterns. The results of the study indicate that the alternative hypothesis (H_1_) regarding the vertical, sagittal, and transverse dimensions is largely accepted.

The vertical measurements revealed no statistically significant difference in the total anterior and lower anterior facial heights between the groups. In contrast, the upper anterior facial height and posterior facial height were significantly higher in the control group. Abuhijleh et al. [22], Treutlein et al. [23] and Doğan et al. [24] reported in their study that the upper anterior facial height was significantly lower in patients with UCLP. The study by Treutlein et al. [23] revealed an increase in lower anterior facial height in 10-year-old children with UCLP. However, there was no statistically significant difference compared to the control group. In their study, Doğan et al. [24] reported that, in patients with UCLP, the backward rotation of the mandible resulted in an increase in anterior facial height, while the posterior facial height decreased correspondingly. The same study revealed that an increase in lower anterior facial height was a secondary reaction to the increased mandibular rotation [24]. The findings of this study are in line with those of the aforementioned studies.

The assessment of maxillofacial morphology in individuals with UCLP is of considerable importance for the development of appropriate treatment protocols [6]. When studies in the literature are examined, different techniques and methods have been used to evaluate craniofacial structures in individuals with UCLP. These include lateral cephalometric radiographs, posteroanterior radiographs, 2D and 3D photographs, optical surface scans, CT (computed tomography), and CBCT [19,22,23,25,26,27]. In the presented study, nasal width was found to be significantly greater in individuals with UCLP compared to the control group. This situation may lead to aesthetic concerns and functional disorders in patients. A multidisciplinary approach is important for the treatment of these patients. The aim of treatment is to meet functional and aesthetic needs [28].

In this study, patients with UCLP who underwent the same operation and healthy individuals were compared using CBCT. The data obtained show that there are significant morphological deviations in the skeletal features of the midface. In this study, it was found that facial width and sagittal facial depth in individuals with UCLP were significantly reduced compared to the control group. Another study using CBCT examined the transversal craniofacial morphology of patients with UCLP. Buyuk et al. [25] reported that facial width was greater in the control group than in patients with UCLP, but there was no significant difference between the groups. In this study, although the biorbital width was found to be greater in the control group than in the study group, the difference was not statistically significant. These results are consistent with the findings from other studies [16,25].

A review of the literature reveals that studies have been conducted to compare cranial base dimensions in order to ascertain whether there is abnormal development in the cranial bases of individuals with UCLP and to determine whether these abnormalities affect midface development [22,29,30]. Goyenc et al. [29] reported that there was no significant difference in cranial base dimensions between the UCLP group that underwent surgery and healthy individuals. In another study, Abuhijleh et al. [22] divided the control and study groups into subgroups according to growth periods. They reported that the length of the anterior cranial base and posterior cranial base in patients with UCLP was shorter in all growth periods compared to the control group [22]. Liu et al. [30] reported that there was a significant shortening in the length of the posterior cranial base in patients with UCLP who underwent surgery compared to the control group. The findings of this study indicate that individuals with UCLP exhibit significantly shorter anterior and posterior cranial base lengths than those in the healthy non-cleft control group. The results of this study lend support to the view that cranial base lengths are affected by the formation of scar tissue following surgical intervention and by intrinsic tissue defects that occur during the developmental process.

Numerous studies have reported that midface development is influenced by surgical operations and the resultant scar tissue, with attention focusing on the nasomaxillary complex due to the maxilla being the primary source of the deformity [21,31,32]. In the conducted study, the maxillary length in patients with UCLP was found to be significantly shorter compared to healthy controls, and there is consistency between these findings and the results of other studies [21,24,30,31]. Additionally, in our study, maxillary transverse width was found to be significantly lower in patients with UCLP. In our study, the inclusion of UCLP individuals who did not undergo any additional surgical procedures in the study group and the control group who did not receive any orthodontic treatment allowed the identification and inclusion of craniofacial changes that occur during the natural course of growth.

The mean age at the time of surgical repair in repaired UCLP patients varies depending on the surgical protocol. Primary lip repair can be performed before 15 weeks [33] and up to 2 years of age [34], and hard palate repair can be performed from 3 months [35] to 4 years [36]. Chen et al. [34] performed hard palate repair with the Millard lip closure technique before the age of two and with the two-flap palatoplasty technique before the age of 3. They reported that early surgical repair may affect the sagittal maxillary growth pattern in UCLP patients. Liao et al. [36] reported that, as a result of hard palate repair performed with the von Langenbeck technique on a 4 year old, the basal maxilla was displaced forward and prevented the anteroposterior development of the maxillary dentoalveolus. These previous studies show that different surgical techniques applied at different times are effective in the development of the maxilla. In the present study, a significant decrease in maxillary width and length was observed in individuals with UCLP who underwent lip repair before one year of age and palate repair before three years of age compared to healthy individuals. Therefore, it would be beneficial to plan the surgical technique and the timing of surgery in consideration of clinical outcomes.

It has been stated that, in contrast to the maxilla, the direction of the development and morphology of the mandible may be more cleft-related and will not be affected by surgical procedures [37]. Silva et al. [38] divided children with UCLP between the ages of four and seven into two groups and compared their facial morphologies. These two groups were treated with two different surgical operations. It showed that different surgical techniques had no effect on mandibular growth and morphology.

In our study, consistent with the literature, while mandibular length was found to be shorter in surgically treated patients with UCLP compared to healthy controls, no statistically significant difference was observed between the two groups [30,31]. The results of the present study indicate that mandibular width is significantly shorter in individuals with UCLP, which may be attributed to malformation or a shortened cranial base. In addition, the fact that maxillary and mandibular anterior alveolar heights are higher in patients with UCLP compared to the control group. Although not statistically significant, this indicates that the anterior alveolar height is not affected by lip closure surgery.

In the present study, the maxillary sagittal length was found to be shorter in the study group compared to the control group, while no significant difference was observed in the mandibular length. In contrast, the maxillary and mandibular transverse widths were found to be smaller in the study groups, indicating a transverse constriction of the maxillomandibular complex in the UCLP group. In the vertical dimension, N-ANS height was significantly lower in the study group due to nasomaxillary developmental delay, whereas no significant difference in the N-Me height was observed between the groups. Similarly, no significant difference was found between groups in the maxillary and mandibular anterior dentoalveolar heights. Thus, it has been revealed that orthodontic treatment planning is necessary to support both the vertical and transverse development of the maxilla in individuals with UCLP while maintaining control of the total anterior facial height. In addition, due to the similarity in total anterior facial height between normal and UCLP individuals, we recommend maintaining control of this height due to the downward and backward rotation of the mandible caused by early maxillary protraction treatments using face masks in UCLP patients [39,40].

The difficulties encountered by researchers in the evaluation of craniofacial morphology in individuals with CLP have been regarded as significant limiting factors in previous studies [14,37]. Among these limitations, the most obvious is the small sample size, along with others such as a wide age range distribution, the lack of distinction between operated and non-operated individuals, the surgeon’s experience, the application of different surgical techniques, and the inclusion of individuals with various types of clefts in the same study [14,25,26,27,28,29,30,31,37]. Nonetheless, the present study provides substantial findings that are made possible by the high power of the existing sample size, which allows clinicians to conduct a comprehensive evaluation of orthodontic and surgical collaboration in diagnosis and treatment planning.

## 5. Conclusions

Individuals with CLP need to be treated carefully with a multidisciplinary approach to ensure functional and aesthetic rehabilitation. For this purpose, it is important to understand the craniofacial growth and morphological characteristics of individuals with CLP. In conclusion, our study compared individuals with UCLP who underwent the same surgical procedure with a control group of healthy non-cleft individuals using CBCT images, revealing significant morphological deviations. The vertical upper anterior facial height and posterior facial height were found to be significantly lower in the UCLP group than in the control group. The study revealed that the midface width in the transverse direction and the facial depth in the sagittal direction were insufficient in individuals with UCLP. Furthermore, in the transverse direction, the maxillary and mandibular widths were found to be narrower in individuals with UCLP, while the maxilla was shorter in the sagittal direction, with no differences observed in the mandible.

## Figures and Tables

**Table 1:** Anatomical points and reference planes used in measurements.

Landmark	Abbreviation	Description
Unilateral		
Sella	S	Geometric midpoint of the sella turcica in sagittal, axial, and coronal views.
Nasion	N	The most anterior point of the frontonasal suture in axial and sagittal images and the midmost point in coronal images.
Anterior nasal spine	ANS	The most anterior point of the anterior nasal spine in the sagittal view and the midpoint in the axial view.
Posterior nasal spine	PNS	The most posterior point of the posterior nasal spine in sagittal view and the midmost point in axial and coronal images.
Prosthion	Pr	It is the lowest and most anterior point of the alveolar crest between the upper central incisors in the middle oxal plane.
Subspinal	A	In the sagittal view, it is the deepest point of the bone tissue concavity between the supadentale and the anterior nasal spine. In the axial view, it is the most anterior and middle point of the premaxilla. In the coronal view, it is the midpoint between the root tips of the upper central incisors.
İnfradental	Id	It is the highest and most anterior point of the alveolar crest between the lower central incisors in the middle oxal plane.
Pogonion	Pog	The most anterior point of the mandibular symphysis in the sagittal image, the most anterior and middle point in the axial image, and the lowest point in the coronal image.
Menton	Me	The lowest point of the mandible in sagittal and coronal images and the middle point in axial images.
Gonion_center_	Go_cen_	The midpoint of the line connecting the right and left gonion points.
Articular_center_	Ar_cen_	The midpoint of the line connecting the right and left articulating points.
Bilateral		
Gonion_right_-Gonion_left_	Go_r_-Go_l_	The point where the bisector of the angle formed by the lines tangent to the mandibular corpus and ramus intersects the mandible in the sagittal image, the most posterior point of the corpus in the axial image, and the lowest point of the ramus in the coronal image.
Articular_right_-Articular_left_	Ar_r_-Ar_l_	The point where the condyle head intersects with the skull base in the sagittal image, and the most convex point of the condyle head in the axial image.
Jugale_right_-Jugale_left_	J_r_-J_r_	The point where the zygomatic arch intersects the tuber maxilla on the jugal process.
Ag_right_-Ag_left_	Ag_r_-Ag_l_	Lateral and inferior edges of the antigonial process.
Om_right_-Om_left_	Om_r_-Om_l_	Inner orbital edges closest to the mid-oxal plane.
Zf_right_-Z_fleft_	Zf_r_-Zf_l_	These are the intersection points of the zygomaticofrontal suture and the orbit.
Zm_right_-Zm_left_	Zm_r_-Zm_l_	It is the center of the zygomatic arch root.
Nc_right_-Nc_left_	Nc_r_-Nc_l_	These are the points located in the widest and outermost region of the nasal cavity in the frontal section.
Reference Planes		
Frankfort plane	FH	It is the plane passing through the orbitale and porion points.
Midsagittal plane	MSP	Passing through Basion and S, perpendicular to the FH plane.
Coronal plane	CP	It is the plane perpendicular to the FH and MSP planes, passing through the basion.

Notes: r:—right, l—left, center(Cen)—the midpoint of the line connecting two left and right points.

**Table 2:** Gender and age distribution between the study and control groups.

	Study Group (*n*)	Control Group (*n*)	*p* Value
**Gender**			
**Female**	15	15	1 ^a^
**Male**	30	30	
Age (year, mean ± SD)	14.69 ± 3.95	14.46 ± 3.65	0.775 ^b^

^a^ Results of the Pearson chi-square test comparing the distribution of the genders; ^b^ Results of the Student *t*-test comparing age distribution of the groups. Data are expressed as mean ± standard deviation. *n*—sample size, SD—Standard deviation.

**Table 3:** Comparison of measurements of the study group and control group obtained with CBCT. All values are calculated in millimeters (mm).

CBCT	Study Group			Control Group			
**Vertical Measurements**	**Min.** **(mm)**	**Max.** **(mm)**	**Mean ± SD** **(mm)**	**Min.** **(mm)**	**Max.** **(mm)**	**Mean ± SD** **(mm)**	***p* Value**
N-Me	93.85	131.85	111.56 ± 9.21	97.39	132.87	113.59 ± 8.30	0.275
N-ANS	36.51	59.43	47.73 ± 4.53	40.10	58.89	50.71 ± 3.60	**0.001**
ANS-Me	51.36	80.72	64.71 ± 7.58	51.08	78.66	63.77 ± 6.40	0.523
S-Go_cen_	56.54	85.48	70.30 ± 7.45	64.48	94.16	76.93 ± 6.79	**<0** **.** 001 *
**Facial Measurements**							
Zf_r_-Zf_l_	86.20	105.99	93.63 ± 4.39	83.84	104.45	95.17 ± 4.59	0.107
Zm_r_-Zm_l_	101.82	134.61	113.90 ± 5.99	103.77	139.89	117.17 ± 6.26	0.013 *
Nc_r_-Nc_l_	19.83	34.92	25.48 ± 2.98	18.18	25.96	22.16 ± 1.97	<0.001 *
Om_r_-Om_l_	12.93	25.43	19.90 ± 2.62	13.45	23.25	18.89 ± 1.95	0.040 *
Ar_cen_-A	61.88	81.42	72.83 ± 5.28	70.05	93.45	79.66 ± 5.75	<0.001 *
**Cranial Measurements**							
S-N	58.80	76.79	65.40 ± 4.15	58.37	76.48	67.58 ± 3.92	0.012 *
S-Ar_cen_	25.72	42.97	34.51 ± 3.85	29.16	43.73	36.62 ± 3.48	0.008 *
**Maxillary Measurements**							
ANS-PNS	28.05	55.46	42.65 ± 6.60	38.97	58.86	50.53 ± 4.08	<0.001 *
ANS-Pr	8.65	22.45	16.52 ± 3.22	9.92	22.89	16.47 ± 3.10	0.935
J_r_-J_l_	58.49	76.07	66.03 ± 4.37	60.82	81.68	68.45 ± 5.20	0.019 *
**Mandibular Measurements**							
Go_cen_-Pog	52.40	97.35	67.93 ± 7.50	58.91	79.64	68.57 ± 4.89	0.637
Id-Me	23.00	37.73	29.11 ± 3.94	23.63	33.99	28.69 ± 2.77	0.557
Ag_r_-Ag_l_	72.51	92.40	82.45 ± 4.56	73.26	97.48	85.12 ± 5.06	0.010 *

* *p* < 0.05, SD—standard deviation; Notes: N-Me—total anterior facial height, N-ANS—upper anterior facial height, ANS-Me—lower anterior facial height, S-Go_cen_—posterior facial height, Zf_r_-Zf_l_—biorbital width, Zm_r_-Zm_l_—facial width, Nc_r_-Nc_l_—nasal width, Om_r_-Om_l_—interorbital width, Ar_cen_-A—facial depth, S-N—anterior cranial base, S-Ar_cen_—posterior cranial base, ANS-PNS—maxillary length, ANS-Pr—maxillary anterior alveolar height, J_r_-J_l_—maxillary width, Go_cen_-Pog—mandibular length, Id-Me—mandibular anterior alveolar height, Ag_r_-Ag_l_—mandibular width.

## Data Availability

Most of the data generated or analyzed are included in the article. The remaining datasets used and/or analyzed during the current study are available from the corresponding author upon request.
